# Effect and Mechanism of *Tricholoma matsutake* Extract on UVA and UVB Radiation-Induced Skin Aging

**DOI:** 10.4014/jmb.2411.11085

**Published:** 2025-02-18

**Authors:** Lu Hu, Zhenhai Huang, Jiyu Weng, Chujie Huang, Lanyue Zhang

**Affiliations:** 1 SHE LOG (Guangzhou) Biotechnology Co., Ltd., Guangzhou 510000, P.R. China; 2 School of Biomedical and Pharmaceutical Sciences, Guangdong Provincial Key Laboratory of Plant Resources Biorefinery, Guangdong University of Technology, Guangzhou 510000, P.R. China

**Keywords:** *Tricholoma matsutake*, photo-aging, inflammation, antioxidant, anti-aging

## Abstract

Ultraviolet (UV) radiation often causes skin aging, inflammation, cancer and other related skin diseases. In this study, the main components of *Tricholoma matsutake* extract (TME) were identified using UPLC-Q-TOF-MS, and their anti-photoaging effects were assessed through UV-induced cell and animal models. The key components identified were D-mannitol (27.41%), DL-malic acid (14%), alginate (12.5%), isoleucine (4.82%), and phenylalanine (4.31%), all of which played roles in anti-aging and UV protection. TME (50-100 mg/ml) significantly alleviated UVA/UVB-induced erythema and wrinkles in mice. Pathological staining showed that TME suppressed UV-induced epidermal hyperplasia (*p* < 0.05), reduced collagen damage (*p* < 0.01), and decreased mast cell infiltration (*p* < 0.01), while down-regulating inflammatory markers such as IL-6, IL-1β, and TNF-α. TME also upregulated type I collagen (COL-1). Flow cytometry results demonstrated that high-dose TME inhibited UV-induced apoptosis and reduced reactive oxygen species (ROS) in HaCaT cells (*p* < 0.05). Immunofluorescence and scratch migration assays showed that TME promoted PPAR-α expression, reduced inflammation, and supported skin repair (*p* < 0.01). Transcriptomic and metabolomic analyses indicated that TME regulated inflammation-related signaling pathways, helping to prevent skin aging. TME is a promising natural product for skin care and treatment of oxidative stress and inflammation-related diseases.

## Introduction

Skin aging is a very common but complex process. There are many factors leading to skin aging, which can be mainly divided into two categories: exogenous factors and endogenous factors. Endogenous factors, namely natural aging, are mainly affected by endocrine and genetic factors, such as deoxyribonucleic acid (DNA) damage, free radical production, telomere shortening, decreased collagen synthesis ability, and cell apoptosis. Exogenous factors are environmental factors, such as external Ultraviolet (UV) radiation, toxic substances in the environment, cigarette smoke, etc., [1]. Exposure of human skin to excessive UV radiation often leads to sunburn, inflammation, erythema, photoaging and even skin cancer. Adverse external environment can also aggravate human skin aging [2-4]. Due to the destruction of the ozone layer and environmental pollution, the UV rays that reach the surface are gradually increasing [5]. UV radiation from sunlight is a recognized environmental hazard that can cause sunburn, pigmentation, inflammation, immunosuppression, and damage to dermal connective tissue, destroying normal skin tissue and leading to photoaging and skin cancer [6].

Photoaging is one of the major factors that contribute to skin aging and caused by UV radiation, which accelerates skin aging and collagen degradation, and causes skin inflammation, skin cancer, and other diseases [7]. The main clinical symptoms include skin wrinkles, erythema, uneven skin color, telangiectasia [8]. Regarding pathogenesis, UV radiation is the best studied pathogen of photoaging. UV rays cause changes in skin structure by stimulating oxidative damage and proinflammatory pathways. They are the central point of photoaging detection [9]. UV radiation can stimulate skin molecules to produce a large amount of reactive oxygen species (ROS), which can react with a variety of intracellular molecules, such as proteins and lipids. In addition, ROS can promote the release of epidermal growth factor, interleukin and tumor necrosis factor [10]. After UV irradiation promotes the generation of reactive oxygen species, it also activates NF-κB, a transcription factor that upregulates the expression of proinflammatory cytokines and growth factors such as interleukin (IL)-1, tumor necrosis factor (TNF), and epidermal growth factor (EGF) [11]. What’s more, UV radiation can activate several matrix metalloproteinases (MMPs), such as MMP-1, MMP-2, and MMP-9, which can degrade collagen in the dermis, leading to loss of skin elasticity [12]. UV radiation can cause histological changes in the damaged skin, such as the fragmentation of elastin and collagen, the increase of glycosaminoglycans and proteoglycans, the increase of inflammatory cells (such as mast cells, eosinophils, and monocytes) in photoaged tissue, and the up-regulation of melanin production [13-16]. The antioxidant capacity of the skin is an important defense mechanism against environmental damage, so there is increasing interest in using plant antioxidants to reduce UV damage to the skin [17-21].

*Tricholoma matsutake* (*T. matsutake*), a natural edible fungus, is considered a valuable food ingredient due to its unique aroma and taste. It has been reported that *T. matsutake* contains a variety of active ingredients including polysaccharides, flavonoids, proteins, amino acids and so on [22, 23]. Meanwhile, *T. matsutake* also reveals excellent biological activities such as antioxidant, anti-cancer, anti-inflammatory, anticoagulant, anti-radiation, anti-fatigue and immunomodulatory [24-26]. *T. matsutake* peptide has been shown to be immunoactive and can improve cellular inflammation by inhibiting the release of pro-inflammatory factors such as IL-6, IL-7β, TNF-α, inducible nitric oxide synthase (iNOS) and cyclooxygenase (COX)-6. These anti-inflammatory effects may be due to the inhibition of MAPK and NF-κB activation [27-29]. *T. matsutake* extract (TME) has a toxic effect on cancer cells, and the activity of cancer cells is significantly reduced after treatment with TME, indicating the antitumor activity of *T. matsutake* [30]. Our previous research has shown that the combination therapy of TME with bakuchiol and ergothioneine can regulate exogenous stimuli and cancer-related signaling pathways, preventing skin aging [31]. Additionally, Deng *et al*.'s study indicates that TME reduces COX-2 expression by inhibiting NF-KB activity, thereby alleviating skin damage caused by photoaging [32]. However, research on photoaging induced by UVA and UVB, simulating real sunlight, is lacking. The results of this study demonstrate that TME has a protective effect against skin damage induced by combined UVA and UVB exposure in mice. *T. matsutake* is a natural product with potential in the development of treatments for skin inflammation and cancer-related diseases.

This paper aims at exploring the protective effects of TMEs on UVA and UVB radiation-induced skin photoaging *in vitro* and *in vivo* studies. TMEs are applied topically on the UVA and UVB-irradiated dorsum skin of mice. The thickness, number of mast cells, and collagen fibers of these irradiated skin are evaluated, and the levels of inflammatory factors in these irradiated skins are tested. TMEs are used for UVA and UVB-irradiated HaCaT cells, and the viability and migratory capability are observed. Meanwhile, HaCaT cells are applied to evaluate the expression of PPAR, apoptotic cells and ROS content. Besides, the mechanisms of TMEs on UVA and UVB radiation-induced skin photoaging are evaluated via metabolomics and transcriptomics. Overall, *T. matsutake* has considerable potential as a natural ingredient in food, cosmetic and pharmaceutical applications.

## Materials and Methods

### Materials and Chemicals

According to the morphological description presented in the China species. The *T. matsutake* was collected in September 2022 from Sichuan Province, China, and the samples were identified by Professor Liu Nian (Zhongkai University of Agriculture and Engineering, China). Some of the samples were deposited in the Institute of Natural Medicine and Green Chemistry (Guangdong University of Technology, China) as reference specimens (No. 2023-116T). Ethanol, 2, 4-dinitrochlorobenzene, propylene glycol, formic acid, glacial acetic acid, phosphomolybdic acid, and xylene were purchased from Aladdin Chemical Reagent Co., Ltd., (China). Drying oven, DHG-9023A, Suzhou Jiangdong Precision Instrument Co. Ltd., (China). Thermostat water bath, HH-2, Changzhou Guohua Electric Appliance Co., Ltd., (China). Rotary evaporator, RE-52AA, Shanghai Yarong Biochemical Instrument Factory (China). Macroporous resin column, D101, Cangzhou Bao 'en adsorption material Technology Co., Ltd.,(China). Cell experimental reagents were purchased from Gibco. United States; The instruments and reagents used for immunohistochemistry included: Dehydrator Donatello, purchased from DIAPATH; Implantation machine JB-P5, purchased from Wuhan Junjie Electronics Co., Ltd., (China) Pathological slicer RM2016, purchased from Shanghai Leica Instruments Co., Ltd.; Freezing table JB-L5, purchased from Wuhan Junjie Electronics Co., Ltd., (China) Tissue spreading machine KD-P, purchased from Jinhua City Kedi Instrument Co., Ltd., (China). Oven GFL-230, purchased from Tianjin City Laiborui Instrument Co., Ltd., (China). Cryostat CRYOSTAR NX50, purchased from Thermo Fisher Technology (China) Co., Ltd.; adhesive slide G6012-1, purchased from Servicibio; cover glass 10212432C, purchased from Jiangsu Shitai Experimental Equipment Co., Ltd., (China). upright optical microscope NIKON ECLIPSE E100, purchased from Nikon Japan; imaging system NIKON DS-U3, purchased from Nikon Japan; Absolute ethanol 100092683, xylene 10023418, purchased from Sinopharm Chemical Reagent Co., Ltd.; environmentally friendly dewaxing solution G1128, universal tissue fixing solution G1101, hematoxin-eosin (H&E) HD constant dyeing kit G1076, toluidine blue dyeing solution G1032, purchased from Servicibio; neutral gum 10004160, glacial acetic acid 1000218, purchased from Sinopharm Chemical Reagent Co. Ltd., (China) PPARα antibody, CY3 labeled goat anti-rabbit, FITC-labeled donkey anti-goat, COL-I, IL-1β, IL-6, TNF-α, etc. were all purchased from Affinity Co. (America) the ultra-high resolution quadrupole combined electrostatic field orbital trap liquid mass spectrometer used in metabolomics was purchased from Thermo Fisher Scientific (USA), instruments used in the transcriptome include: crushing grinder TL-48R, purchased from Shanghai Wanbai Biotechnology Co., Ltd. China; Small centrifuge ABSON MiFly-6, purchased from Hefei Eibenson Scientific Instruments Co. Ltd., (China), High-speed benchtop refrigerated centrifuge Eppendorf 5424R, purchased from Eppendorf (Germany), electrophoresis instrument DYY-6C, purchased from Beijing City Liuyi Instrument Factory (China), Ultramicro spectrophotometer NanoDrop2000, purchased from Thermo Fisher Scientific. Bioanalyzer Agilent 5300, purchased from Agilent (USA), Fluorescence Quantifier Qubit 4.0, purchased from Thermo Fisher. PCR instrument T100 Thermal Cycler, purchased from BIO-Rad (USA), sequencer NovaSeq X Plus was purchased from illumina (USA), reagents used include: QIAzolLysis Reagent, available from Qiagen (Germany). RNA Purification Kit, purchased from Shanghai Meiji Biomedical Technology Co. Ltd., (China), Biowest Agarose, purchased from Biowest (Spain), Illumina® Stranded mRNA Prep, Ligation (constant), purchased from Illumina. NovaSeq Reagent Kit, available from illumina, the rest of the experimental reagents were purchased from Macklin (China).

### Extraction

Fresh *T. matsutake* was selected and de-mixed, thinly sliced with a knife, and transferred to a drying oven for drying. The dried *T. matsutake* was inspected, including visual inspection, nasal smell, and chemical test (moisture). After passing the quality inspection, the *T. matsutake* (50 g) was transferred to a flask for extraction in the thermostat water bath, heated to reflux by deionized water (100 ml) as solvent at 80°C for 2 h, and filtered under reduced pressure. Then the filtered extract was concentrated under reduced pressure by rotary evaporator and chromatographed on a macroporous resin column, further refined and de-mixed. The chromatographed solution was concentrated under reduced pressure by rotary evaporator.

### Components of Extracts Determined by UPLC-Q-TOF-MS

UPLC-Q-TOF-MS (Thermo, Ultimate 3000LC, Q Exactive HF) instrument analytical platform. C18 column (Zorbax Eclipse C18, 1.8 m × 2.1 mm × 100 mm). The following were the chromatographic separation conditions: the flow rate was 0.3 ml/min, the sample size was 5 μl and the column temperature was 30°C. Compositions of the mobile phases A and B were water and 0.1% formic acid, respectively. The auto sampler's temperature was 4°C, and the injection volume was 2 ml. Heater temperature in the positive mode was 325°C, sheath gas flow was 45 arb, auxiliary air flow was 15 arb, and purge gas flow was 1 arb. Capillary temperature was 330°C, electrospray voltage was 3.5 KV, and the S-Lens RF level was 55%. Heater temperature in negative mode was 325°C; Purge gas flow rate was one arb, auxiliary air flow rate was 15 arb, sheath gas flow rate was 45 arb, and electrospray voltage was 3.5 KV. 330°C for the capillary temperature and a 55% S-Lens RF level. Scanning modes include data-dependent second-order mass spectrometry (dd-MS2, TopN=10) and first-order Full Scan (m/z 100–1500); 70,000 (for primary mass spectrometry) and 17,500 (for secondary mass spectrometry) resolutions. High energy collision dissociation (HCD) is the collision mode.

### Animals

Twenty five SPF KM mice, male, 5 weeks old, with clean backs free of erythema, purchased from the Experimental Animal Center of Guangdong Province, were housed at 22°C with a 12-h light/dark cycle for four days. TMEs were dissolved in saline. The 25 mice were randomly divided into five groups: control group, model group, *T. matsutake* extract 50 mg/ml group (TME-L), *T. matsutake* extract 75 mg/ml group (TME-M), and *T. matsutake* extract 100 mg/ml group (TME-H). The concentrations were chosen based on previous studies [31, 32] and with reference to the catalog of cosmetic raw materials already used in China. On the day before the experiment, the mice were depilated on the back with an area of approximately 4 cm × 4 cm. Except for the control group, the remaining mice received UVA and UVB combined irradiation (UVA = 1,000 mJ/cm^2^; UVB = 100 mJ/cm^2^) once a day for a week. Starting from the 8^th^ day, the experimental mice were treated every other day with UVA and UVB radiation, followed by the topical application of 200 μl of 50 mg/ml, 75 mg/ml and 100 mg/ml TME on the hairless area of the back, once a day. UVA and UVB radiation can induce erythema, particularly when the skin is exposed to excessive UV radiation. Additionally, the inflammatory response, allergic reactions, or skin infections caused by the modeling procedure may also lead to erythema or the appearance of erythematous spots. The control group mice received no treatment. Subsequently, the mice were euthanized using cervical dislocation, and the back skin tissues were fixed in 4% paraformaldehyde and frozen for histological staining with hematoxylin-eosin (HE), toluidine blue (TB), Masson's trichrome, immunohistochemical stainings.

### Hematoxylin and Eosin (HE) Staining

The thickness of mouse epidermis was assessed using a Hematoxylin and eosin kit. Hematoxylin and eosin kit was used to evaluate the epidermal thickness of mice. The paraffin section was soaked in xylene, absolute ethanol, and 75% ethanol in turn. The slices were immersed in 95% alcohol, absolute alcohol, and xylene for 5 min., and the tissues were fully dehydrated and transparent. After drying, neutral gum was dropped to seal the slices. Image acquisition was performed after neutral gum drying, and the epidermal thickness was measured by Image Pro Plus software.

### Toluidine Blue Staining

The number of mast cells in the skin tissue was calculated by Toluidine blue staining. Paraffin sections were deparaffinized into water: sections were sequentially placed in xylene 20 min-xylene 20 min-absolute ethanol 5 min-absolute ethanol 5 min-75% ethanol for 5 min. and washed with tap water. Toluidine blue staining: animal tissue sections were soaked in staining solution for 2 to 5 min., rinsed with water, micro differentiated with 0.1%glacial acetic acid, and terminated by running water rinsing, and the degree of differentiation was controlled under the microscope. After washing with tap water, the slices were placed in the oven to dry. Transparent seal: The sections were placed in a clean xylene transparent box for 10 min and sealed with neutral glue. Scan slides with a fully automated digital slide scanner to acquire images and analyze using Image Pro Plus.

### Masson Staining

Paraffin sections dewaxed to water: Place the slices into environmentally friendly dewaxing solution I for 20 min-environmentally friendly dewaxing solution II for 20 min-anhydrous ethanol I for 5 min-anhydrous ethanol II for 5 min-75% alcohol for 5 min., wash with tap water; re-warm and fix frozen slices: Remove the frozen slices from-20°C refrigerator and restore to room temperature, fix them with tissue fixative solution for 15 min., and rinse with running water; immerse the slices in Masson A solution overnight, and rinse with running water; Cut the slices into a dye solution mixed with Masson B solution and Masson C solution in equal proportions, dip for 1 min, wash with tap water, and the differentiation solution differentiated for a few sec., wash with tap water; dip the slices into Masson D solution for 6 min., rinse with tap water; dip the slices in Masson E solution for 1 min.; without washing, slightly drain and directly add Masson F solution for dyeing for 2-30 s; rinse the slices with 1%acetic acid to differentiate, and dehydrated two cylinders of absolute ethanol; transparent mount: The slices were placed in the third cylinder of absolute ethanol for 5 min., xylene was transparent for 5 min, and the slices were sealed with neutral gum. Scan slides with a fully automated digital slide scanner to acquire images and analyze using Image Pro Plus.

### Immunohistochemical Staining

The paraffin sections of mouse skin were stained with immunohistochemical staining, and the sections were deparaffinized to water and immersed in citric acid antigen repair solution for antigen repair. After washing, 3%H_2_O_2_ was added drip to block endogenous peroxidase. After blocking, the corresponding primary and secondary antibodies were added drip to incubation, and then color development and hematoxylin counterstained were performed. Finally, the slices were gradually dehydrated in 75% alcohol, 85% alcohol, and absolute ethanol, and then placed in xylene for 5 min. After drying, the slices were sealed with neutral gum. Image-Pro Plus software was used for measurement, and graphpad prism software was used for data analysis and drawing. The measured indexes in this experiment were IL-6, IL-1β, TNF-α and COL-I.

### Cell Viability Was Measured by MTT Assay

HaCaT cells were seeded in 96-well plates at a density of 60,000 cells per ml, 100 μl per well. After 18-24 h, the cells were attached to the wall and treated with different concentrations of TME (100, 200, 50, 25, 12.5 μg/ml), respectively, and the cells were incubated for 24 h. The blank group and the control group were added with the same amount of PBS or medium, respectively, each group had 5 replicates. After 24 h, the prepared MTT solution was diluted 10-fold with culture medium, 100 μl was added to each well, and each group was wrapped with tin foil and incubated in an incubator in the dark. Each group was wrapped with tin foil and incubated in an incubator away from light. Four hours later, the MTT diluent or PBS was discarded with tissue, and 100 μl DMSO was added. After shaking, the absorbance (570 nm) of Mezan was measured by enzyme marker (Multiskan FC, Thermo Fisher Scientific), and the survival rate of the cells was calculated.

### Cell Immunofluorescence Assay

After being digested and harvested by trypsin, 1 × 10^5^ HaCaT cells were seeded into 6-well plates with glass slides for standard culture. Upon reaching 80%~90% confluency, the cells were treated with different concentrations of TME based on their groupings (TME-H: Contains 200 μg/ml TME, TME-M:Contains 100 μg/ml TME, TME-L:Contains 50 μg/ml TME) for 24 h. The growth medium was removed, and the cells were washed thrice with PBS. Fixed with 4% paraformaldehyde for 30 min, and permeabilized using 0.3% Triton X-100 (1 ml per well) for 20 min. at room temperature. Blocked with 0.5% normal goat serum, followed by incubation with primary PPAR antibody solution at 4°C overnight. The cells were then washed thrice with PBS before incubation with FITC-labeled secondary antibody solution for 60 min. at 37°C. Finally, DAPI was added dropwise onto a cover glass slide, stained for 5 min at room temperature, and washed thrice with PBS. Positive staining for green cytoplasm and blue nucleus was observed. Three films per group were taken, each with 10 high magnification fields (400×). The integrated optical density was analyzed using the Image Pro Plus (IPP) software.

### Scratch Healing Experiment

The scratch healing experiment assesses cellular migration ability. Each HaCaT cells group was cultured in a 6-well plate with a density of 1 × 10^5^ cells per well. Once the cells formed a monolayer at 90% confluence, a 200 μl pipette suction head was used to create scratches in the single-layer cells. The loose cell fragments were then washed with PBS and cultured in serum-free medium for 0 and 24 h. Subsequently, the scratch images were captured under a microscope to observe the migration distance of monolayer cell growth edges which were then used to calculate the scratch healing rate after 24 h of injury.

### Apoptosis Was Detected by Flow Cytometry

Apoptotic cells were identified by flow cytometry using Annexin V-FITC/PI apoptosis detection kit. The 6-well plates were used for cell model and drug administration. After the plates were cultured for 24 h to make the cells stick to the wall, 100 μL PBS was added to each well after discarging the old medium, and then UV irradiation (the dose was determined by MTT assay) and drug administration were performed, and the cells were cultured for 24 h. Staining was performed according to the instructions, the cells were resuspended in 1×Binding Buffer and then stained at room temperature in the dark with 5 μl Annexin V-FITC and 5 μl PI solution for 30 min. The fluorescence intensity was detected by flow cytometry.

### Flow Cytometric Measurement of ROS

In this experiment, Biyuntian reactive oxygen species detection kit was used for detection, and fluorescent probe DCFH-DA was used for the detection of reactive oxygen species, because DCFH-DA can freely cross the cell membrane. Afterwards DCFH and DCF with fluorescence signal will be blocked in the cell, so the intracellular fluorescence signal can be detected by flow cytometry to react with the ROS. Detection of ROS Cells were pelleted, washed twice with fresh PBS, centrifuged to remove the PBS liquid, pelleted again and then transferred to a 2 ml EP tube. A 1:1000 dilution of the probe DCFH-DA in serum free media was loaded onto the cells. The samples were incubated in the dark at 37°C for 20 min., then washed with fresh cell culture media three times to remove the DCFH-DA, and then were analyzed by flow cytometry to determine the intracellular ROS concentration.

### Metabolomics Analysis

The selection of metabolic experimental methods was revised based on previous literature. Weigh 1,000 mg of cryopreserved skin sample and mix it with 1,000 μl of 80% methanol, and homogenize it with 3.2 mm chromium steel beads using a homogenizer (60 HZ, 90 s/2 times); then the mixture was sonicated in ice water for 10 min. The treated mixture was centrifuged at 12,000 rpm at 4°C for 15 min. The supernatant was then filtered using a NestBiotech 0.22 μm filter, and 500 μl of the filtered supernatant was pipetted with a syringe and added to a sample bottle for further analysis. Chromatographic separation of target compounds is carried out through liquid chromatography columns using Raman high performance liquid chromatography. The chromatography setup mainly consists of the following three parts: Agilent 1290 Infinity LC SYS High Performance Liquid Chromatography System (UPC), Separated Hydrophilic Interaction Liquid Chromatography (HILIC) column (25°C), flow rate 0.5 ml/min, injection volume 2 μl. The main components of the mobile phase are A: water mixed with 25 mmol/l ammonium acetate and 25 mmol/l ammonia, and B: acetone. Elution conditions should follow the following principles: 0-0.5 min, 95% B; 0.5 min-7 min, 95%-65% B; 7 min-8 min, 65%-40% B; 8 min-9 min, 40% B; 9 min-9.1 min, 40%-95% B; 9.1 min-12 min, 95% B. AB Triple TON 6600 MS was selected for sample collection, mainly obtaining the primary and secondary spectra of the sample. Conditions for the ESI source are as follows: The ion source gas ratio is 1:60, the ion source gas ratio is 2:60, the current gas is 30, the IonSapary floating voltage is ± 5,500 V, and the positive and negative mode is adopted; the MS (TU) scanning range within the mass-to-charge ratio (m/z) range is 60-1,000 Da, and the product ion scanning range is 25-1,000 Da; the collected data are subjected to secondary MS analysis and high-sensitivity mode is adopted. The aggregation potential of both positive and negative modes is ± 60 V, and the collision energy is (35 ± 15) eV.

### Transcriptome Sequencing

TRIzol was used to extract total RNA from mouse skin samples and assess the purity, concentration, and integrity of RNA samples to ensure that qualified samples are used for transcriptome sequencing. In order to perform transcriptome sequencing of library data, this paper uses the IlluminaHis-eq4000 platform for analysis. Low-quality data was filtered out of the original data by a fast quality control method to ensure data quality. This process involves removing sequences that contain linkers, have over 10% uncertain bases (N), include base A, or contain more than 50% low-quality bases (Q ≤ 20). Ribosomal alignment, sequencing alignment, and reference genome alignment were performed, followed by transcript reconstruction to calculate all genes levels in samples. Sample correlation analysis and differential expression analysis were conducted, labeling genes with FDR < 0 and |log2(FC)| > 1 as significantly different. These analyses were carried out using short sequence alignment tools such as Bowtie2, HISAT2, Stringtie, along with R language and DESeq. The differentially expressed proteins were compared and tested one by one in various terms in the GO database, and the differentially expressed genes were condensed and analyzed by GO. In addition, the sequencing results were compared with the KEGG database, and the differentially expressed genes were functionally annotated and classified.

### Statistical Analysis

The data from each experimental step were analyzed using GraphPad Prism software (version 9.1). The results were presented as the mean ± standard deviation (SD). One-way analysis of variance (ANOVA) was utilized to compare the means across multiple groups, while pairwise comparisons between smaller groups were performed using a two-sided Student's t-test. Multiple measurements were performed with a minimum of three replicates, unless otherwise specified. The statistical significance was determined based on the *p*-value. The results are marked as significant “*” when *p* < 0.05, “**” when *p* < 0.01.

## Results and Discussion

### Chemical Components of TME

**Fig. 1:**
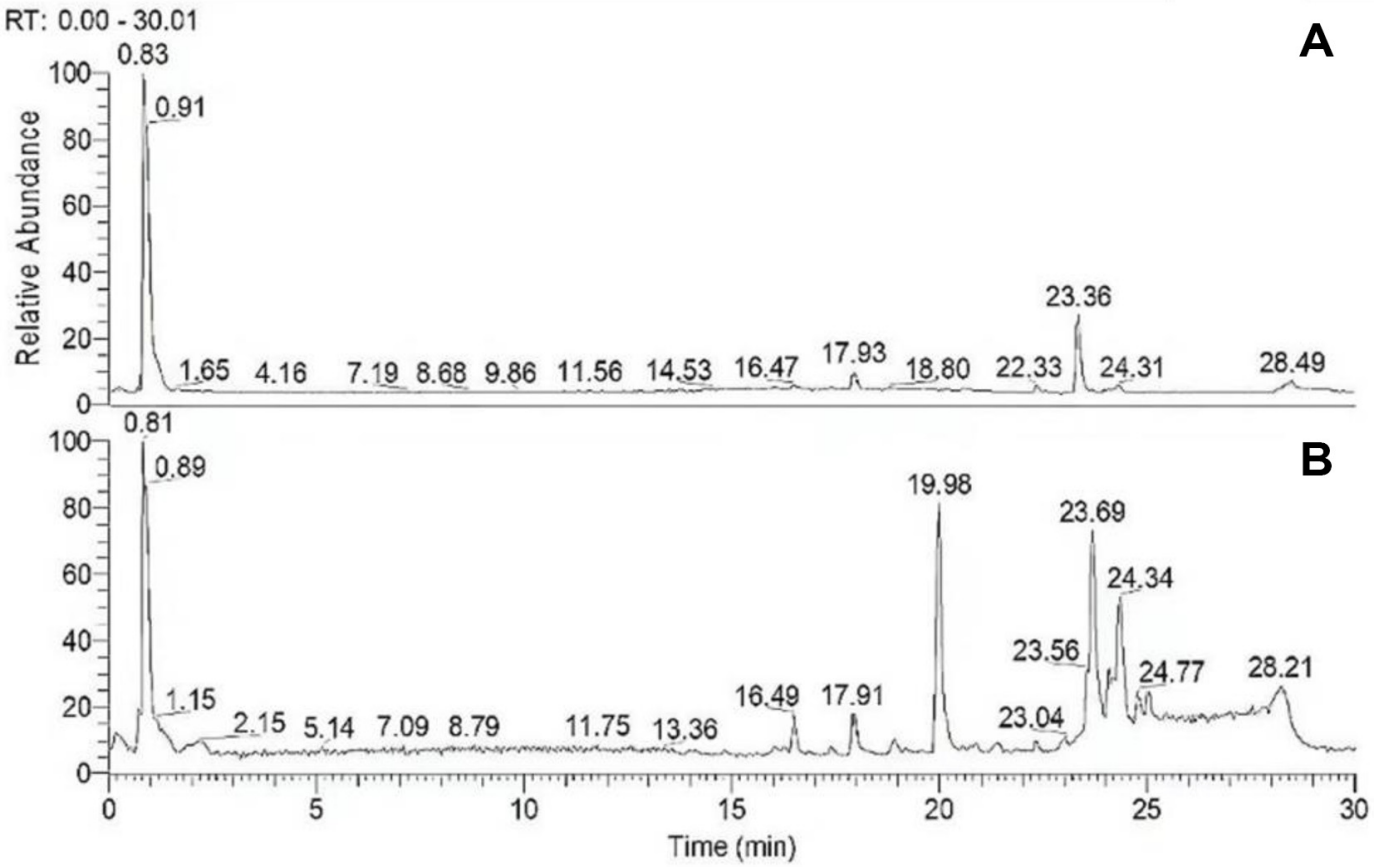
Total ion chromatograms acquired in positive and negative modes of TME. From top to bottom are the total ion maps obtained in positive (A) and negative (B) modes, respectively.

Fig. 1 was the full scan of positive and negative ions of TME. In Table 1, the obtained data were preliminarily processed by Thermo Xcalibur 4.1 software, the exact molecular weight test value of the detected compound was matched with the theoretical value, and was searched in the *mz*-Cloud database of Compound Discoverer, and analyzed and identified. It could be seen from Table 1 that the components with the highest content were sugars and amino acids, accounting for 43.53% and 13.76% of all components. The most abundant compound was D-mannitol (sugar) with a relative content of 27.41%, in addition to 14% DL-malic acid (carboxylic acid), 12.50%alginate (sugar), 4.82% isoleucine (amino acid), 4.31% phenylalanine (amino acid) and 4.15% dextroquinate (carboxylic acid). It was roughly consistent with the ingredients of *T. matsutake* that had been identified [33]. Previous studies had shown that D-mannitol, malic acid and alginate have good potential for scavenging oxidative free radicals [34-36].

### The Restorative or Protective Effect of TME on Photoaged Mice

**Fig. 2:**
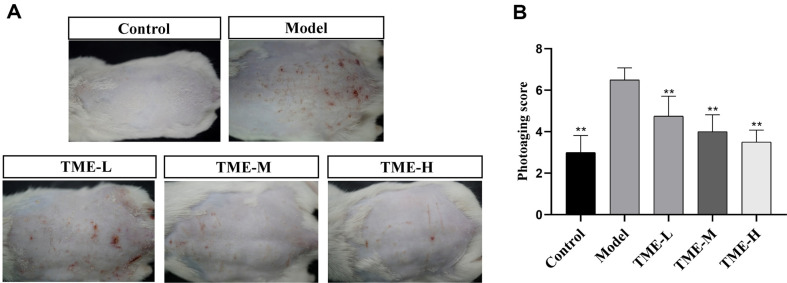
Image and analysis results of skin tissue on the back of mice. (**A**) shows erythema and wrinkles on the back skin of mice after administration. (**B**) shows the statistical analysis results of photoaging scores on the back of mice, **p < 0.01.

The clinical severity of skin lesions in mice was assessed by the formation of skin lesions such as erythema and wrinkles, as shown in Table 2, which were graded as 1 (none), 2 (mild), 3 (moderate), 4 (severe), and 5 (very severe). The sum of the two scores was the final score [37]. As could be seen from Fig. 2A, UV-irradiated model group had more erythema and wrinkles than the control group, while the drug group had significantly fewer wrinkles and erythema than the model group, showing a significant improvement effect. Fig. 2B showed the results of statistical analysis using GraphPad software after the mouse was scored according to the appearance of erythema and wrinkles on the back. The photoaging appearance score of the model group was significantly higher than that of the blank group, while the photoaging score of the back skin of the mice treated with different concentrations of TME was significantly lower than that of the model group, and the results were statistically significant (*p* < 0.05). Therefore, it could be considered that TME in the concentration range of 50 mg/ml-100 mg/ml had a repair or protection effect on photoaging mice.

### The Inhibitory Effect of TME on UV-Induced Epidermal Hyperplasia

**Fig. 3:**
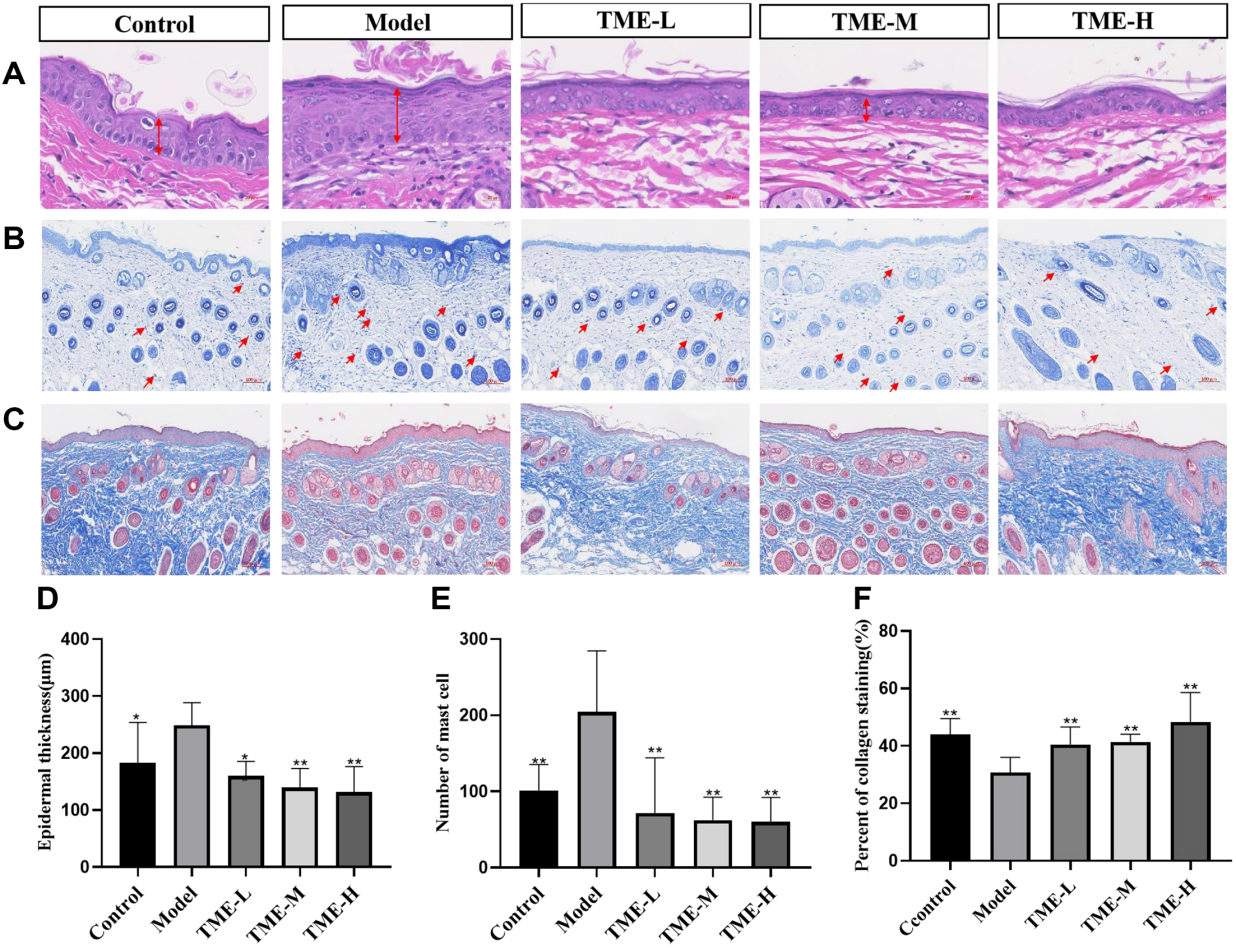
Staining Image of mice Dorsal Skin Tissue. (**A**) Hematoxylin-eosin stained mice back epidermis. (**B**) Stained sections of mice back skin tissue. (**C**) masson stained mice back epidermis, in which muscle fibers are purple red and collagen fibers are blue. (**D**) Representative epidermal thickness for each treatment group. Values are expressed as mean ± SEM. Significance of data was analyzed by one-way ANOVA followed by Dunnett’s test (*p* < 0.05). * indicates significant difference at *p* < 0.05; ** indicates significant difference at *p* < 0.01. (**E**) The number of mast cells in mice skin tissue was counted. Values are expressed as mean ± SEM. Significance of data was analyzed by one-way ANOVA followed by Dunnett’s test (*p* < 0.05). ** indicates significant difference at *p* < 0.01. (**F**) Values are expressed as mean ± SEM. Significance of data was analyzed by oneway ANOVA followed by Dunnett’s test (*p* < 0.05). ** indicates significant difference at *p* < 0.01.

UV irradiation caused skin epidermal hyperplasia, and this experiment assessed the anti-aging ability of *T. matsutake* using epidermal thickness as an indicator [38]. In Fig. 3A and 3D, the epidermis without UV treatment was thin, flat, and regular in shape. Epidermal thickness was significantly increased in UVA and UVB mice compared to non-irradiated controls (*p* < 0.01). The epidermis thickness of mice treated with different concentrations of TME was much thinner than that of the model group. The therapeutic effect was positively correlated with the concentration of TME.

### The Inhibitory Effect of TME on UV-Induced Mast Cell Infiltration

Mast cells originated from pluripotent stem cells in the bone marrow, migrated to tissues to mature, and could produce inflammation [39]. In this study, the anti-inflammatory effect of *T. matsutake* was evaluated by mast cell infiltration. Mast cell infiltration could reflect the severity of cellular inflammation. Fig. 3B and 3E showed that the number of mast cells in the model group after UV irradiation was significantly higher than that in the blank group, while the number of mast cells in each group was reduced after treatment with TME. The inhibitory activity of TME on macrophages was enhanced with increasing concentration, which might reduce the production of pro-inflammatory cytokines.

### The Repair Effect of TME on UV-Induced Collagen Fiber Damage in Skin Tissue

UV radiation could directly damage collagen fibers in the dermis, and Masson staining could distinguish collagen fibers from muscle fibers in the tissue. Therefore, Masson staining was used to evaluate the content and arrangement of collagen fibers in the skin tissue. The evaluation criteria were to calculate the relative area of blue collagen fibers to reflect the repair effect of TME on collagen fibers, and to visually observe whether the collagen fibers were distributed in an orderly manner. In Fig. 3C and 3F, the collagen fibers in the back skin of the blank group were closely and neatly arranged, while the content of blue collagen fibers in the dermis of the model group was significantly reduced, the arrangement was disorderly, and some collagen fibers were broken. The content of collagen fibers in the three treatment groups was higher than that in the model group, and the collagen bundles were arranged neatly and tightly. The results showed that TME could inhibit UV rays damage to collagen in skin tissue.

### The Regulatory Effect of TME on UV-Induced Cytokine Expression in Skin

**Expression of IL-6 in mouse dorsal skin.** It had been shown that skin exposed to excessive UV radiation could cause a series of skin inflammations [40]. The production of interleukin (IL-1β, IL-6) and other inflammatory (TNF-α) cytokines played an important role in skin inflammation.

**Fig. 4:**
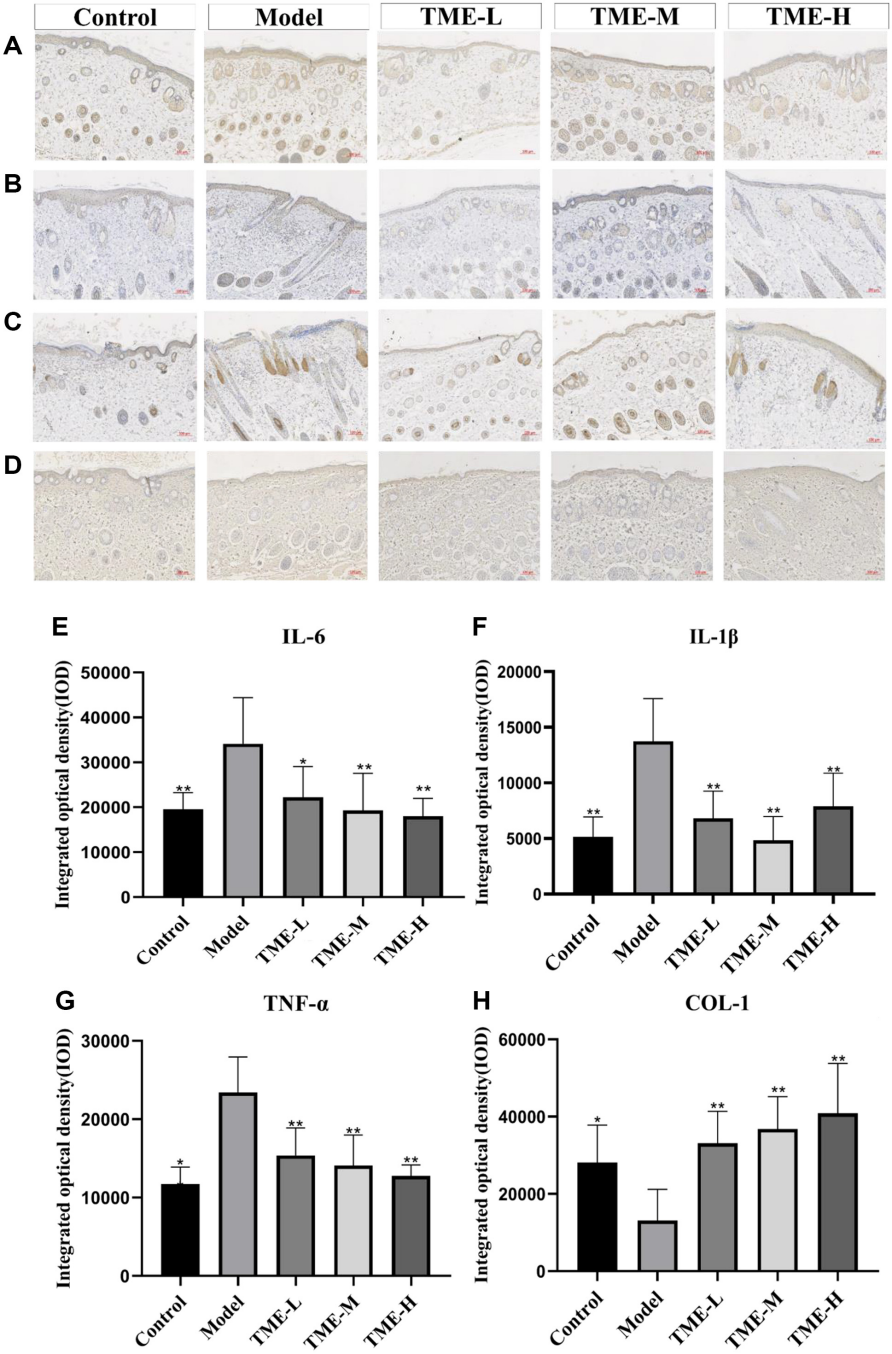
Immunohistochemical Staining Image of Skin Tissue. (**A**) Immunohistochemical staining of IL-6 in mice back skin. (**B**) IL-1β immunohistochemical staining results of mice back skin tissue. (**C**) Immunohistochemical staining of TNF-α in mice back skin. (**D**) COL-1 immunohistochemical staining results of mice back skin tissue. (**E**) IL-6 integrated optical density (**IOD**) values in different groups of mice. Values are expressed as mean ± SEM. Significance of data was analyzed by oneway ANOVA followed by Dunnett’s test (*p* < 0.05). * indicates significant difference at *p* < 0.05; ** indicates significant difference at *p* < 0.01. (**F**) IL-1β integrated optical density (**IOD**) values in different groups of mice. Values are expressed as mean ± SEM. Significance of data was analyzed by one-way ANOVA followed by Dunnett’s test (*p* < 0.05). ** indicates significant difference at *p* < 0.01. (**G**) TNF-α integrated optical density (**IOD**) values in different groups of mice. Values are expressed as mean ± SEM. Significance of data was analyzed by one-way ANOVA followed by Dunnett’s test (*p* < 0.05). * indicates significant difference at *p* < 0.05; ** indicates significant difference at *p* < 0.01. (**H**) COL-1 integrated optical density (**IOD**) values in different groups of mice. Values are expressed as mean ± SEM. Significance of data was analyzed by one-way ANOVA followed by Dunnett’s test (*p* < 0.05). * indicates significant difference at *p* < 0.05; ** indicates significant difference at *p* < 0.01.

In Fig. 4A and 4E, the expression of IL-6 in the model group was significantly up-regulated, and the expression of IL-6 after the treatment with three concentrations of TME was lower than that in the model group, and the treatment effect was concentration-dependent.

**Expression of IL-1β in mouse dorsal skin.** According to Fig. 4B and 4F, the expression of IL-1β in the model group after UV irradiation was significantly higher than that in the blank group, and the expression of IL-1β after the treatment with three concentrations of TME was lower than that in the model group. Therefore, it could be considered that different concentrations of TME could inhibit the expression of IL-1β and inhibit the occurrence of inflammatory response.

**Expression of TNF-α in mouse dorsal skin.** As a pro-inflammatory factor, TNF-α could aggravate the inflammatory response by providing adhesion sites for neutrophils and lymphocytes, leading to tissue damage, vascular dilatation, and increased permeability in the inflammatory site [41, 42]. As shown in Fig. 4C and 4G and, compared with the blank group, the expression of TNF-α in the model group was significantly increased, and the expression of TNF-α was significantly decreased after the treatment with TME solution compared with the model group. The 100 mg/ml TME treatment group showed the strongest down-regulation of TNF-α expression.

**Expression of COL-1 in mouse dorsal skin.** The skin dermis contained a large amount of typeIcollagen [43], and the content of collagen in the skin was detected by immunohistochemistry. Fig. 4D and 4H showed that the content of collagen in the model group decreased significantly after UV irradiation, and the collagen content increased significantly after treatment with the extract of *T. matsutake*. The results showed that the extract of Matsuyoshi has the effect of repairing dermal collagen.

### Cytotoxicity Assessment and Safe Concentration Range of TME

**Fig. 5:**
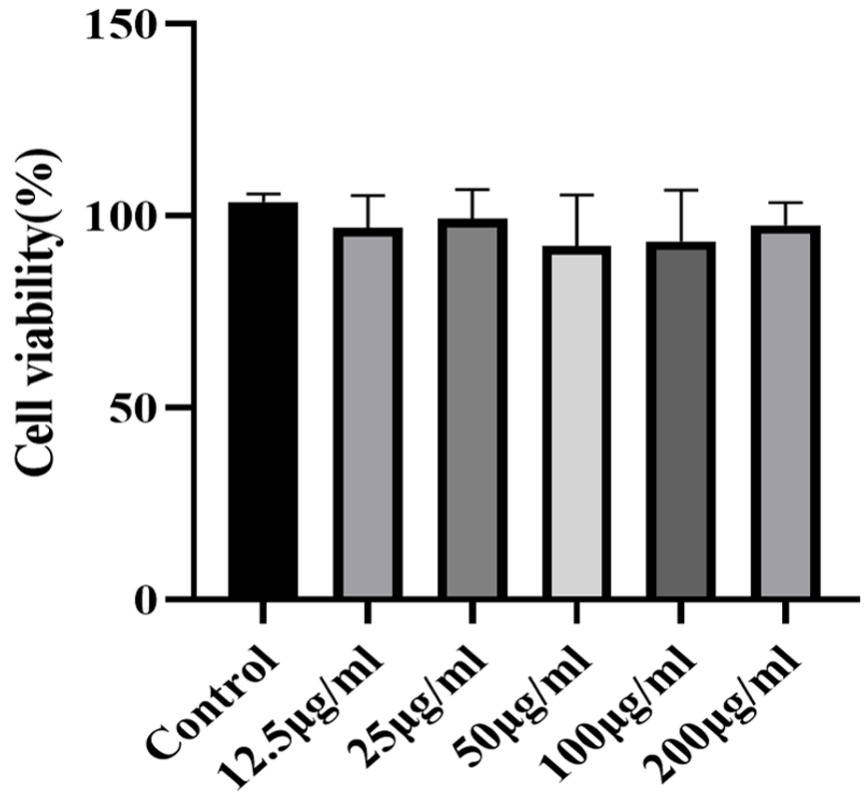
Effect of *T. matsutake* on Cell Vitality of HaCaT.

The purpose of cell MTT assay was to evaluate the survival status, proliferation ability and response of cells to different compounds, thereby providing important reference data for further cell biology research. Fig. 5 showed that compared with the control group, the differences in cell viability after treatment with different concentrations of TME were small, all above 90%, indicating that TME had no cytotoxicity or low cytotoxicity in the concentration range of 12.5-200 μg/ml, which could be used for subsequent experiments.

### Regulatory Effect of TME on UV-Induced PPAR-α Expression

**Fig. 6:**
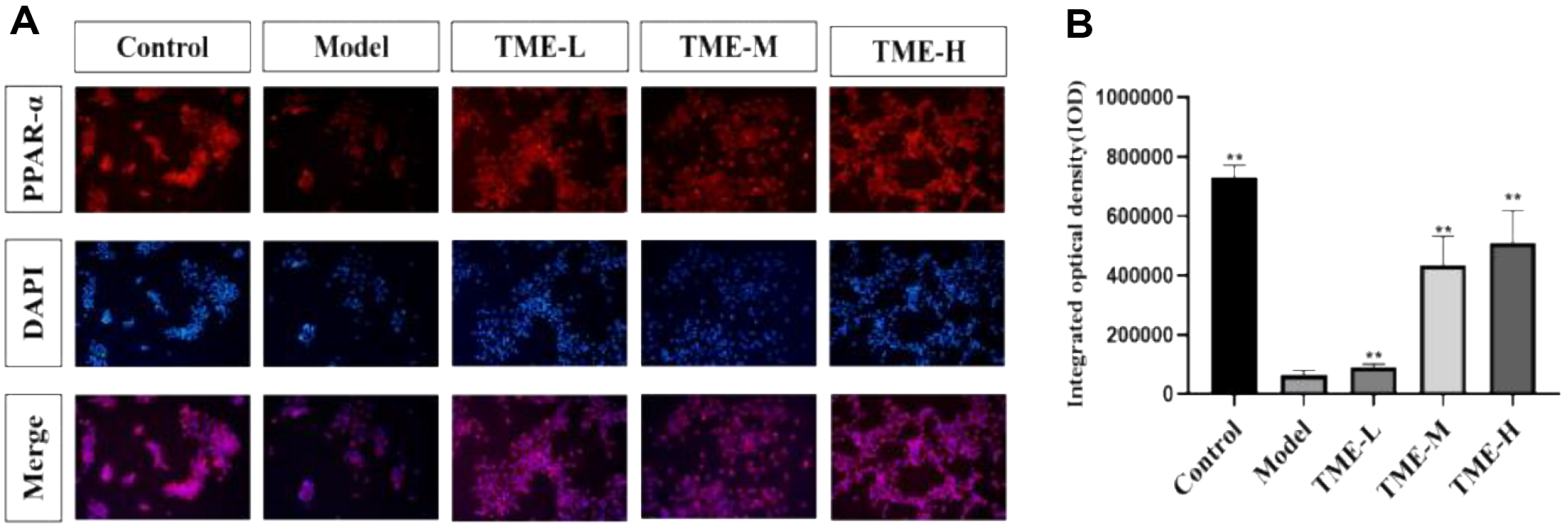
Cellular immunofluorescence staining after different treatment methods. (**A**) Under the microscope, the cell immunofluorescence image shows the expression of PPAR-α in red, the number of DAPI dyes entering the nucleus in blue, and the combination of the two images in purple. (**B**) Detected optical density values of different groups of cells, **p < 0.01.

The nuclear hormone receptor superfamily, which had a key function in controlling immunological and inflammatory responses, included peroxisome proliferator-activated receptors (PPARs) [44]. NF-kB signaling pathway activation inhibition, pro-inflammatory cytokine production inhibition, neutrophil apoptosis induction, and inflammation reduction were all possible effects of PPARs [45]. Fig. 6 showed that UV could significantly reduce the expression of PPAR, and the expression was significantly increased after treatment with different concentrations of TME, among which the up-regulation ability of TME in the 200 μg/ml group was the most obvious. These results indicated that TME could inhibit the occurrence of cellular inflammation, and the therapeutic effect was concentration-dependent.

### TME Promotes Skin Repair through Cell Migration

**Fig. 7:**
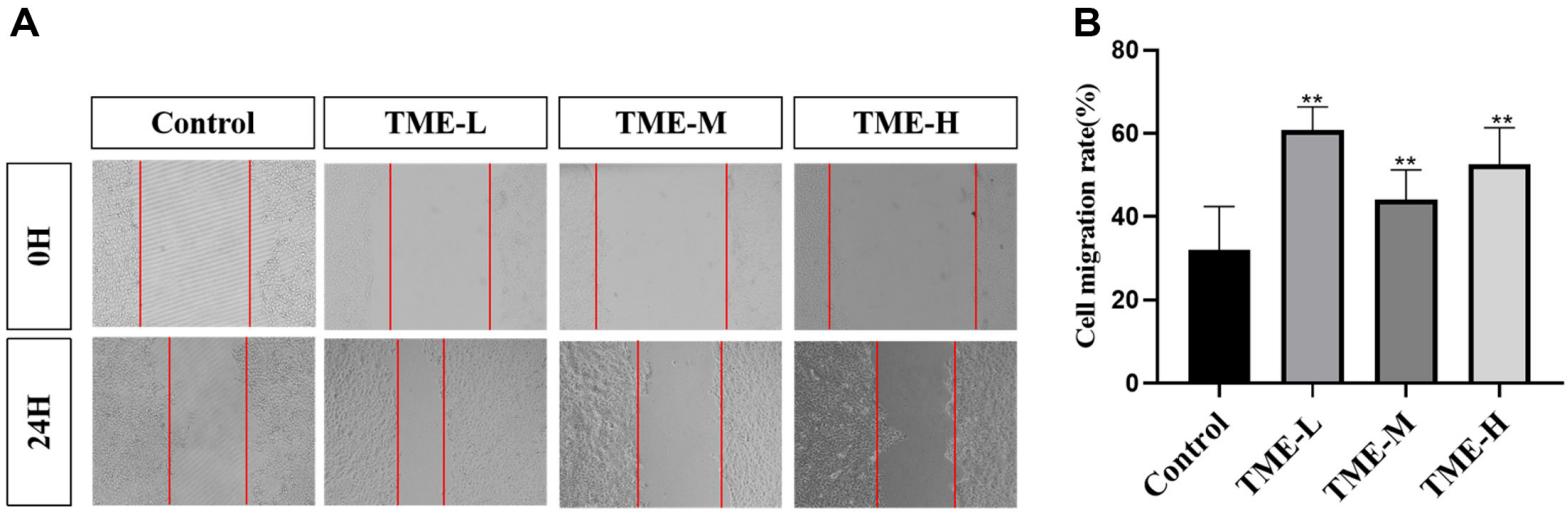
TME promotes cell migration. (**A**) The pictures of scratch wound migration assay,healing due to cell migration of HaCaT observedwithin 0 h and 24 h following scratch wounding. (**B**) Quantitative data from the cell scratch healing assay from (**A**) The mobility of scratch distance at 0H for each group is 100%, **p < 0.01.

The most popular technique for assessing a cell's capacity for migration and healing was the cell scratch test. On the confluent monolayer of cells, a blank patch, known as a scratch, was generated. Cells from the edge would progressively enter the scratch to mend it. Since it resembled the wound-healing process, this assay was widely used to observe the effect of drugs on wound healing *in vitro*. Fig. 7 showed that compared with the blank group, the migration rate of cells treated with drugs for 24 h increased, indicating that TME could play a role in promoting skin repair by promoting cell migration.

### TME Inhibits UV Radiation-Induced Cell Apoptosis

In this study, flow cytometry was used to study the repair or protection effect of *T. matsutake* on UV-induced photoaging. Apoptotic cells would have DNA fragment deletion, and showed weak staining after propidium iodide staining, which was reflected in the histogram of apoptotic peak. The fluorescence intensity of normal cells not in the apoptotic stage was higher than that of apoptotic cells. In addition, due to chromosome changes and autophagosome changed during apoptosis, the forward angular light scattering ability and lateral angular light scattering ability of cells were decreased, which could be used for ring gate analysis.

**Fig. 8:**
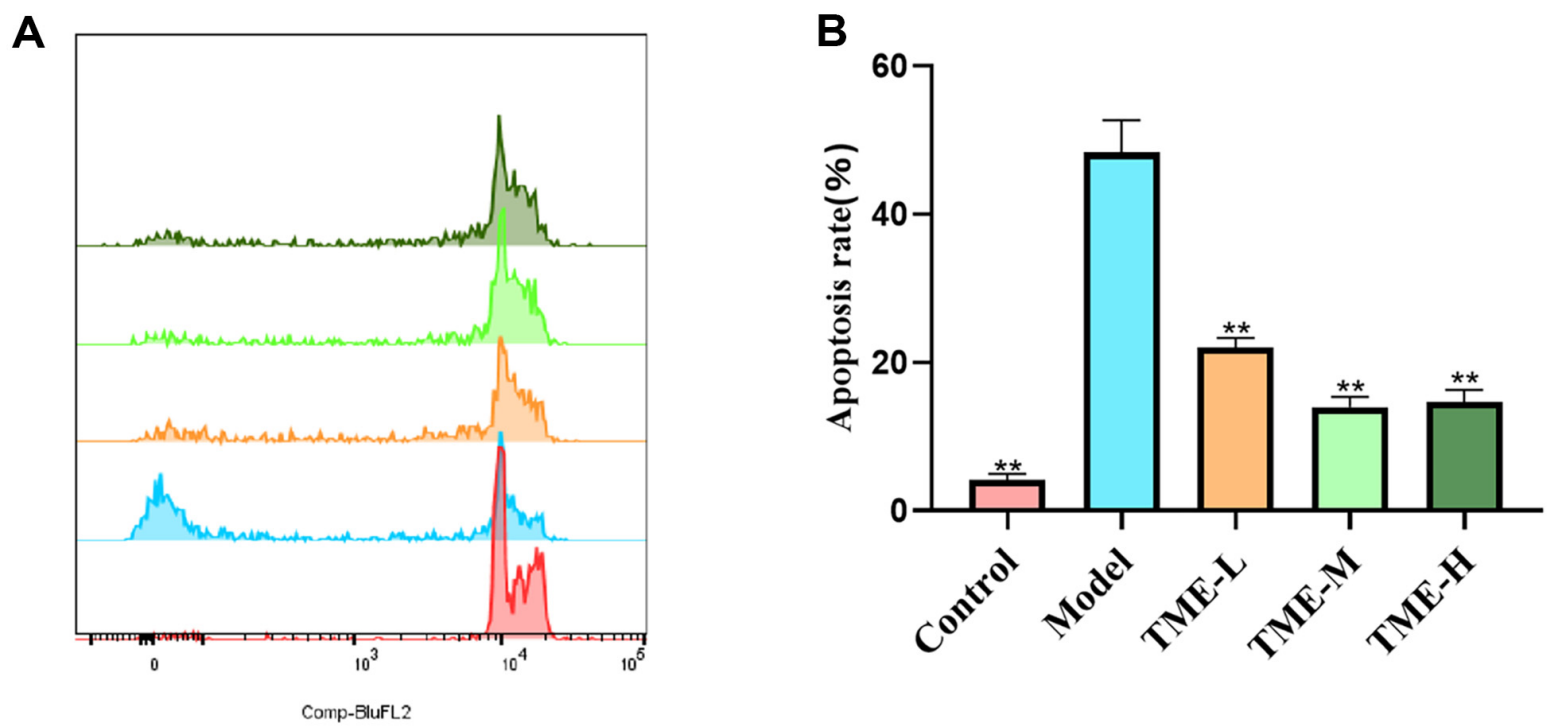
TME inhibits cell apoptosis of HaCaT. (**A**) Flow cytometry of HaCaT cells. The abscissa represents the fluorescence intensity, and the ordinate represents the number of cells at this fluorescence intensity (**B**) Flow cytometry experimental chart of HaCaT cells, **p < 0.01.

Fig. 8A showed histograms of multiple fluorescence channels, with different colors (green, orange, blue, and red) representing distinct fluorescence channels. The horizontal axis was labeled "Comp-HuFL2" and corresponded to specific cellular markers or antigens. The vertical axis quantitatively measured the average fluorescence intensity (MFI) at which cells expressed the marker. The green channel exhibited a peak around 10^3^, indicating a cell population with moderate expression levels. The orange channel showed a more pronounced peak at a lower fluorescence intensity level, around 10^2^. The blue channel demonstrated a narrow distribution suggesting low expression levels, while the red channel displayed a sharp peak indicating a cell population with high expression levels. Fig. 8B showed the statistical results of HaCaT cell apoptosis. The apoptosis rate of the control group was 4.19%, while that of the model group was 48.33%, which was significantly higher than that of the blank group. After treatment with different concentrations of TME, the apoptosis rate of the cells treated with low, medium and high concentrations of TME were 22.0%, 13.97% and 14.67%, respectively, which was significantly lower than that of the model group. Among them, the concentration of 100 μg/ml TME had the best treatment effect, and the results were statistically significant. Therefore, it could be considered that TME could inhibit UV radiation-induced apoptosis.

### TME Clears UV-Induced Intracellular ROS Accumulation

**Fig. 9:**
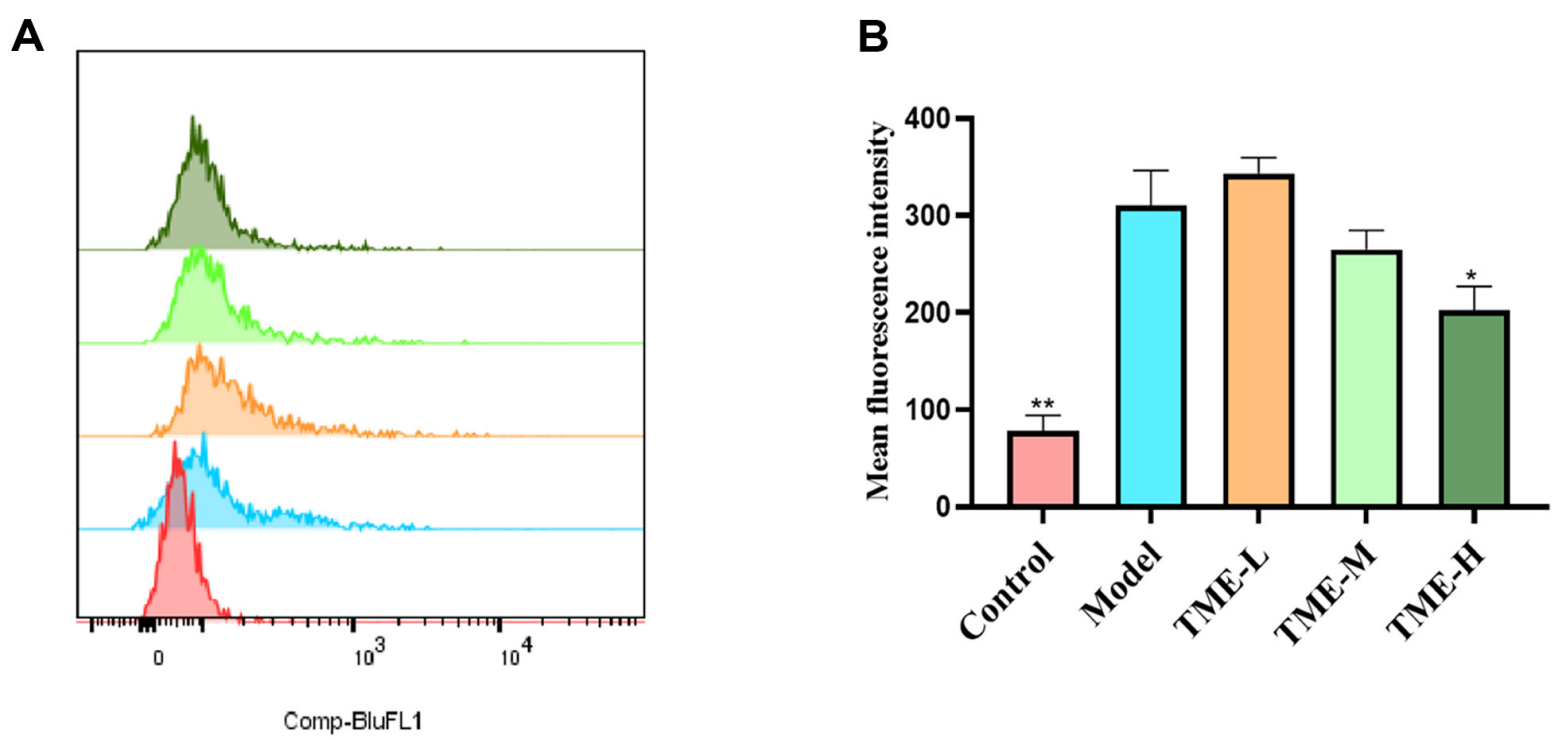
(A) ROS content detected by flow cytometry. The abscissa represents the fluorescence intensity, and the ordinate represents the number of cells at this fluorescence intensity; (B) Statistical analysis of (A) *p < 0.05 and **p < 0.01.

It could be seen in Fig. 9 that after UV irradiation, the fluorescence intensity peak was obviously shifted to the right, but the fluorescence intensity peak was shifted less to the right in the sample group. Fig. 9B showed that the ROS content of cells after UV irradiation was significantly increased, while the ROS content of cells treated with TME was less than that of the model group. However, only the TME-H group was significant, indicating that the TME could remove intracellular ROS.

### The Effect of TME on Regulating Metabolic Pathways Associated with Skin Aging

**Fig. 10:**
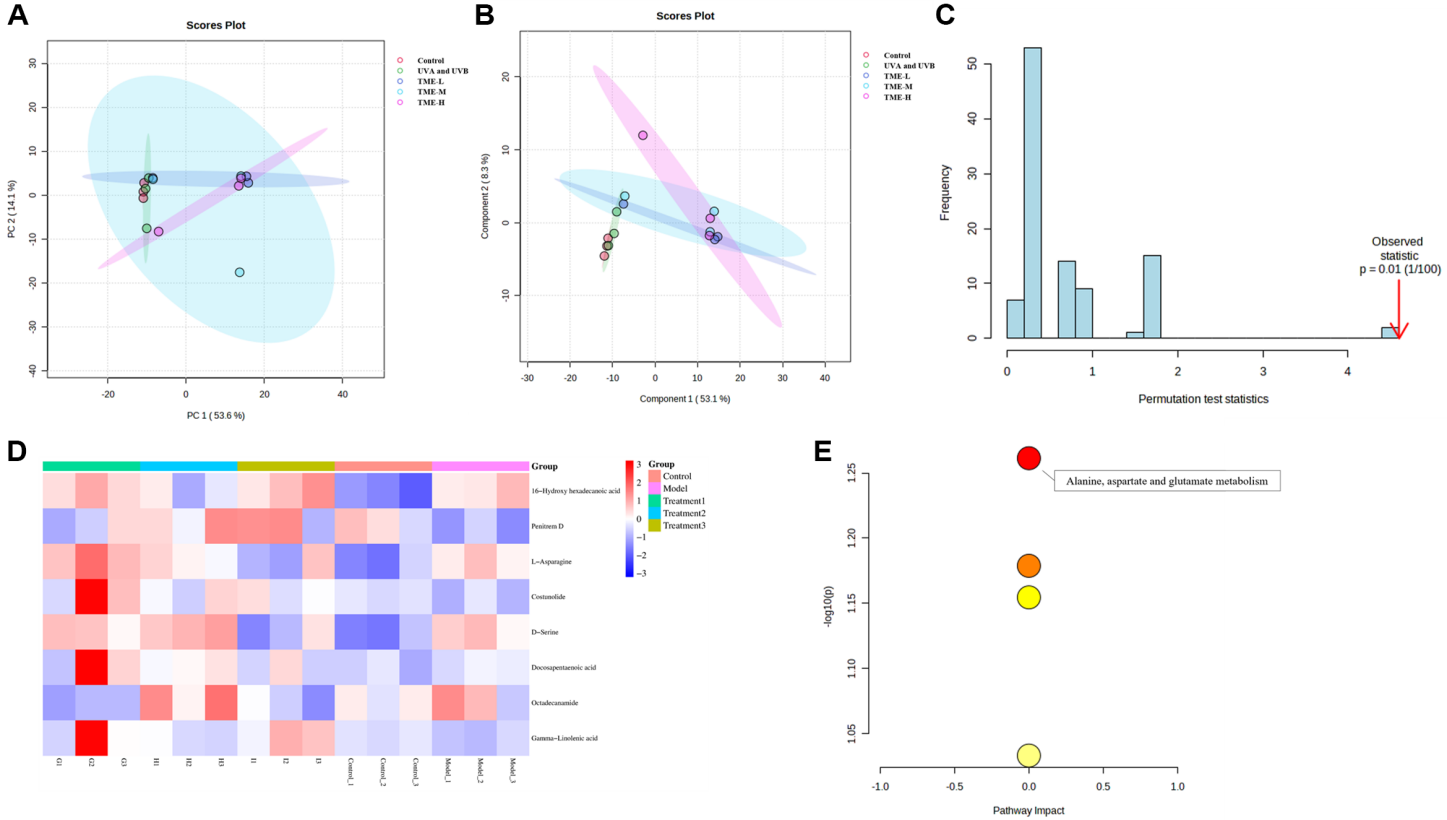
Effects of *T. matsutake*. (**A-B**) The PCA and PLS-DA were used to distinguish the differences in metabolic profiles of different groups, respectively. (**C**) Permutation test was used to verify the accuracy of the PLS-DA model. (**D**) Heat map represented the relative levels of differential metabolites after three groups of EOs treatment. (**E**) Metabolite pathway enrichment map with the classification of metabolites.

In order to study the anti-aging effect of the TME on skin, metabolomic analysis was performed in groups with different TME concentrations. As shown in Fig. 10A, the samples of each group were analyzed with principal components analysis (PCA) to determine the reproducibility of the samples within the group and the differences between the groups. In Fig. 10B, the partial least squares discriminant analysis (PLS-DA) model was used to model the relationship between metabolite expression and the class of the samples. Each group had its own separate clusters, and the samples were effectively separated. The permutation test was used to verify the validity of the PLS-DA model. The results of Fig. 10C after 100 permutations proved that the model has good predictive ability. The clustering heat map of Fig. 10D was based on Variable Important in Projection (VIP ≥ 1) and T-test (*p* < 0.05) to show the identification of 8 drug metabolisms (DMs), including 16-Hydroxy hexadecanoic acid, Penitrem D, L-Asparagine, Costunolide, D-Serine, Docosapentaenoic acid, Octadecanamide and Gamma-Linolenic acid. The majority of these metabolites were reversed following treatment with the TME, suggesting that these metabolites might modulate metabolic disorders to some degree. The 8 DMs were enriched with 4 metabolic pathways in Fig. 10E, mainly involving the four metabolic pathways: Alanine, aspartate and glutamate metabolism, D-Amino acid metabolism, Glycine, serine and threonine metabolism, and Biosynthesis of unsaturated fatty acid. Among them, glutamate, aspartate and glutamate metabolic pathways were significantly changed (*p* < 0.05). According to studies by Francesco Errico *et al*. [46], d-aspartic acid stimulates glutamatergic NMDA receptors (NMDARs), and sustained high d-Asp levels produced NMDAR-dependent neurotoxic effects, leading to precocious nerve inflammation and cell death. Matsutake extract improved the damage caused by photoaging by reducing the expression of d-aspartic acid. In addition, the up-regulation of D-serine and the reduction of dysfunction of glycine, serine and threonine metabolic pathways might be another reason why TME repairs skin photoaging damage. The Alanine, aspartate and glutamate metabolism pathway was significantly altered (*p* < 0.05).

### TME Regulates Gene Expression to Repair Skin Aging via Transcriptomics Analysis

Principal component analysis (Principal Component Analysis, PCA) was a method of statistical analysis of multidimensional data with unsupervised pattern recognition in order to determine the repeatability of the samples and the difference between the samples within the groups. Once the samples had been analyzed by dimensionality reduction, there were relative coordinate points on the principal components. The distance of each sample point represents the distance between samples: the closer the distance was, the higher the similarity between samples, and the better the biological duplication between samples. As shown in Fig. S11A, the samples in each group were clustered together, indicating that the samples in each group were reproducible. All samples were subjected to correlation analysis to assess reproducibility of duplicate biological samples within groups, and also to assess the reliability of differentially expressed genes and ancillary screening of anomalous samples. The Pearson correlation coefficient r (Pearson's Correlation Coefficient) was used as an evaluation of the correlation between biological replicates. Thus, the closer the absolute value of R was to 1, the greater the correlation of the two repeated samples. Good within-group replication was demonstrated in Fig. S11B.

The volcano plot in Fig. S11C ~ 11E showed the total number of genes detected in the differential grouping and the number of significantly up- and down-regulated differential genes. The order plot represented the gene expression fold change and the ordinate indicated the significance level of genes. Green dots represented upregulated differential genes, blue dots represented down-regulated differential genes, and red dots represented non-differentially expressed genes. A corrected *p* value of < 0.05 and a fold change of ≥ 1.2 were used as the screening criteria. Compared with the control group, the 50 mg/ml *T. matsutake* extractive group showed significant downregulation of 682 genes and upregulation of 289 genes in Fig. S11C; 930 genes were significantly downregulated and 514 genes were significantly upregulated in the 75 mg/ml *T. matsutake* extractive group in Fig. S11D; 621 genes were significantly downregulated and 7750 genes were significantly upregulated in the 100 mg/ml *T. matsutake* extractive group in Fig. S11E.

Clustering heatmap showed differentially expressed genes and clustering between samples. The abscissae represented the sample information and hierarchical clustering results, whereas the ordinate represented the difference genes and the hierarchical clustering results. Red colour indicated high expression, and blue colour indicated low expression. As can be seen in Fig. S11F, the significantly expressed genes in the control group were significantly expressed in the treatment group and were most evident in the extractive group of 100 mg/ml *T. matsutake*.

### TME Regulates Skin Aging-Related Pathways through Gene Function Analysis

Gene Ontology (GO) was a standard international classification scheme for the function of genes. It was a database established by the Gene Ontology Consortium, and as such aims to establish a language vocabulary standard that was applicable to across a variety of species, describe and define gene and protein functions, and could be updated with further research. GO consists of three parts: the molecular function, the biological process, and the cellular component. The vertical axes represented secondary GO classification, and the horizontal axes represented gene expression levels.

Kyoto Encyclopedia of Genes and Genomes (KEGG) was a comprehensive database that integrates information about the genome, biological pathways, diseases, drugs, chemicals, and other information. KEGG organically combined genomic information with high level functional information to provide a systematic analysis of the big data generated by genome sequencing and other high throughput experimental technologies. Where the vertical axis represented the KEGG pathway and the top horizontal axis represented the number of genes/transcripts in the pathway being compared, corresponding to different points on the line; The horizontal axis below represented the significance level of enrichment, corresponding to the height of the column. The lower the Padjust value, the larger the s-value log10 (Padjust), and the more significantly enriched the KEGG pathway.

Fig. S11G ~ S11I showed that the GO genes are mainly enriched in binding, biological regulation, cellular process, and cell part in all the three treatment groups as compared to the control group.

**Fig. 12:**
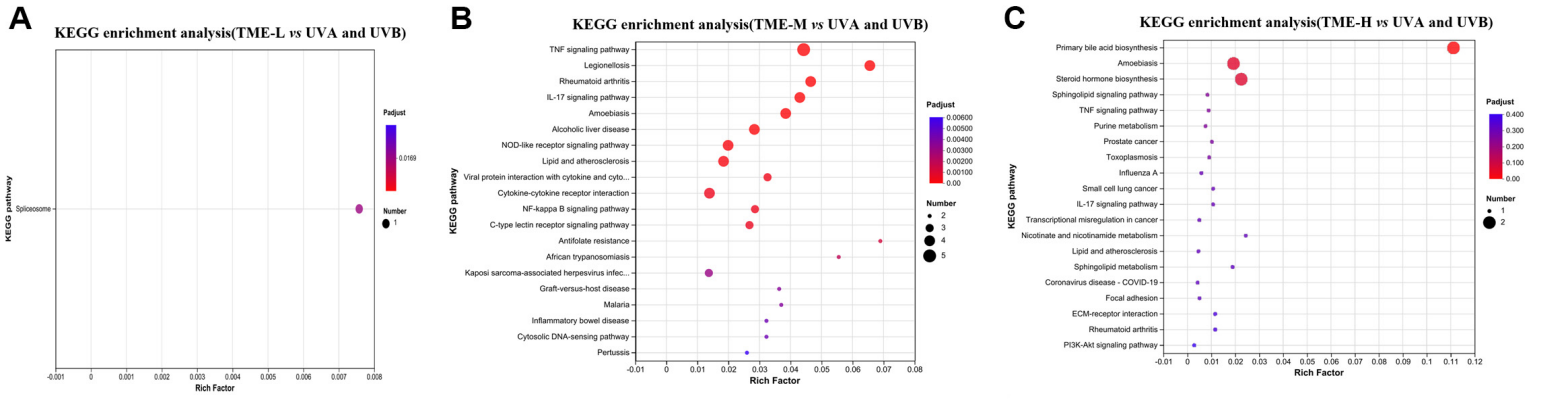
Results of KEGG enrichment analysis of 20 differentially affected genes. (**A**) TME-L compared to the model group. (**B**) TME-M compared to the model group. (**C**) TME-H compared to the model group.

It could be seen from Fig. 12 that KEGG in the control group enriched 20 information pathways related to metabolism, environmental information processing, cellular processes and human diseases. The active ingredients of *T. matsutake* could reduce and inhibit UV damage to skin cells through cytokine receptor interactions, TNF signaling pathway, NF-kappa B signaling pathway L, IL-17 signaling pathway, PI3K-Akt signaling pathway and other pathways. TNF was a cytokine that was involved in inflammatory responses, apoptosis and other processes. TNF mediates signal transduction through cell membrane receptors (such as TNFR1 and TNFR2) and activates multiple signal pathways, including NF-kappa B and PI3K-Akt, etc., which was consistent with the results of enriched differential genes. NF-kappa B could be activated by TNF, leading to nuclear translocation of NF-kappa B and regulating gene transcription. IL-17 was a member of a family of cytokines that was involved in inflammatory regulation and immune responses. It could regulate inflammatory responses by activating NF-kappa B and other pathways. The PI3K-Akt signaling pathway played a key role in the regulation of cell survival, proliferation and metabolism. Akt could regulate NF-κB signaling by phosphorylating IKKα and Tpl2 [47].

## Discussion

*T. matsutake* is a rare edible fungus widely distributed in East Asia and Northern Europe. It is highly regarded not only for its distinctive aroma and flavor but also for its potential medicinal properties, which have attracted the attention of researchers. Recent studies have revealed that *T. matsutake* contains various bioactive compounds with antioxidant, anti-inflammatory, and anti-aging effects, particularly in the context of UV-induced photoaging. Photoaging is a major factor in skin aging, with UV radiation causing a range of age-related processes, including skin damage, inflammation, and collagen degradation. Key components in *T. matsutake*, such as polysaccharides, polyphenols and peptides, have been shown to effectively mitigate UV-induced skin aging. Studies have shown that two novel alkali-extracted polysaccharide fractions, TM-APS-1 and TM-APS-2, isolated from *T. matsutake*, exhibit significant antioxidant activity in a concentration-dependent manner [48]. Meanwhile, polyphenols extracted from *T. matsutake* have been confirmed to exhibit remarkable antioxidant and anti-inflammatory effects by reducing reactive oxygen species (ROS) levels in UVB-exposed mice, upregulating the activity of glutathione peroxidase (GSH-Px), superoxide dismutase (SOD), catalase (CAT), and glucose-6-phosphate dehydrogenase (G6PDH), and inhibiting the expression of IL-1, IL-6, IL-8, and TNF-α [32]. Moreover, *T. matsutake* peptides significantly ameliorate the production of inflammatory cytokines by inhibiting the expression of COX-2, iNOS, IKK-β, p-IκB-α, and p-NF-κB, as well as the activation of the NF-κB/COX-2 pathway [49]. The synergistic effects of these natural compounds make *T. matsutake* a promising natural anti-aging substance. With the growing concern about the health of the skin and the rapid development of anti-photoaging products, there is a strong potential for natural extract products [50].

In this study, the active ingredients are determined through the extraction of *T. matsutake*, and the compounds contained are alcohols, polysaccharides, organic or inorganic acids, and amino acids, with the highest content of mannitol, malic acid, alginate, isoleucine, and phenylalanine. Among them, polysaccharide compounds such as alginate have antioxidant and anti-inflammatory potential, and can effectively repair UV-induced skin photoaging [51]. The results of the pathological section assay demonstrate that TMEs have the effect of inhibiting UV-induced epidermal proliferation, significantly improving mast cell infiltration, and significantly reducing the degradation of collagen fibers in the dermis and protecting and increasing collagen (COL-1). TMEs inhibit oxidative stress-induced inflammation by down-regulating the expression of inflammatory cytokines IL-6, IL-1β, and TNF-α. Further studies showed that UV-induced HaCaT cells were treated with TMEs in the range of 50-200 μg/ml with increased migration rate, and decreased cell apoptosis rate, indicating that TMEs have potential application in promoting the proliferation and epidermal repair of photoaged cells. The upregulation of PPARs expression and the decrease of intracellular ROS content indicate that the repair mechanism of TMEs on photoaged skin is related to the reduction of oxidative stress damage and inflammation expression. Analysis of metabolome and transcriptome results show that TMEs can reduce the metabolic dysfunction of alanine, aspartate and glutamate metabolism, and glycine, serine and threonine metabolism to repair photoaged skin.

In summary, TME has a protective and restorative effect on UV-induced photoaged skin, which has great potential in the development of skin-care products. Although metabolomics and transcriptomic analysis results have been performed, the pharmacological mechanism of anti-photoaging effects of specific TMEs components and specific targets such as TNF signaling pathway need to be further elucidated in this study.

## Figures and Tables

**Fig. 11:**
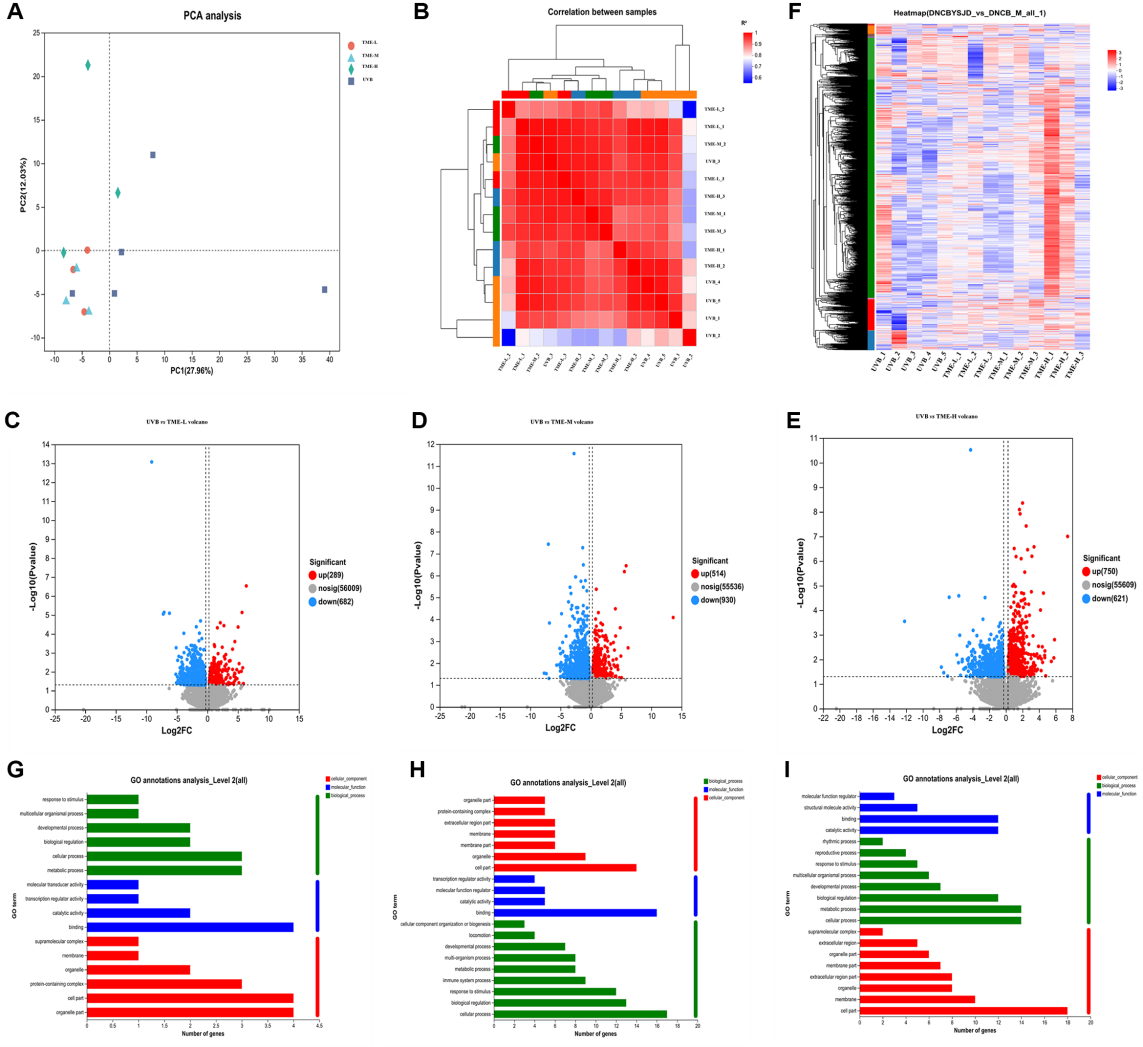
Skin transcriptome analysis. (**A**) Two-dimensional principal component analysis of skin tissues of photoaged mice. (**B**) Heat map of inter-sample correlation. (**C**) TME-L compared to the model group. (**D**) TME-M compared to the model group. (**E**) TME-H compared to the model group. (**F**) Differential gene clustering heatmap. (**G**) TME-L compared to the model group. (**H**) TME-M compared to the model group. (**I**) TME-H compared to the model group.

**Table 1:** Identification of TME by MS/MS spectra obtained in Electrospray ionization (ESI)+ and ESI- modes.

NO	Tentative identification	Fragment ions (m/z) [M+H]+	Fragment ions (m/z) [M+H]-	Relative content/%	RT/min
1	γ-Aminobutyric acid	60.08143,104.10716		0.07	0.908
2	Choline	60.08144,104.10719		2.99	0.914
3	L(+)-Ornithine	70.06567,116.07063		0.67	0.917
4	Gluconic acid		75.00858,129.01917,195.05080	0.30	0.933
5	α,α-Trehalose		59.01377,71.01373,89.02430	12.50	0.934
6	L-Threonine	56.05018,74.06056		0.26	0.939
7	D- (+)-Mannose		59.01375,71.01369,89.02427	0.69	0.94
8	4-Guanidinobutyric acid	86.06043,87.04442,146.09218		0.11	0.945
9	D-Mannitol		71.01370,181.07159	27.41	0.948
10	D-Carnitine	162.11226		0.72	0.948
11	Adenine	136.06158		0.10	0.952
12	D-Serine	60.04503		0.99	0.956
13	D-(-)-Quinic acid		191.05595	4.15	0.961
14	Asparagine	74.02415,87.05564		0.16	0.965
15	L-Glutamic acid	84.04477		3.32	0.972
16	Trigonelline	138.05475		0.03	0.976
17	DL-Malic acid		71.01373,115.00352	14.00	0.994
18	Shikimic acid		73.02935,93.03441	0.04	0.994
19	D-(-)-Glutamine	84.04478,130.04937		0.91	0.998
20	Adenosine	136.06157		2.63	0.999
21	Betaine	118.08625		1.90	1.004
22	Fumaric acid		71.01374	0.14	1.009
23	Glutaconic acid		85.02934	2.08	1.04
24	Citric acid		111.00858,191.05594	1.49	1.055
25	DL-Lactic Acid		89.02429	0.05	1.074
26	L-Pyroglutamic acid	84.04482		1.60	1.079
27	*cis*-Aconitic acid		85.02935	0.23	1.082
28	Nicotinamide	80.04994,123.05530		0.11	1.092
29	Nicotinic acid	80.04996,124.03934		0.27	1.101
30	Succinic acid		73.02937	0.15	1.21
31	L-Isoleucine	86.09681		4.82	1.404
32	Pyrogallol		125.0242	0.03	1.631
33	*L*-Phenylalanine	120.08074		4.31	2.27
34	Indole-3-acrylic acid	118.06512,146.05975		0.56	4.375
35	Abscisic acid	151.07622,219.13887		0.03	16.294
36	15-epi Prostaglandin A1	337.07474		0.2	20.778
37	9,10-Dihydroxy-12-octadecenoic acid	183.13881,201.11308		0.22	21.318
38	9-Oxo-10(*E*),12(*E*)-octadecadienoic acid	67.05479,71.08605,277.21555		0.11	22.031
39	Cafestol	317.20804		0.03	22.084
40	2,3-dihydroxypropyl 12-methyltridecanoate	57.07057,71.08607		0.10	23.048
41	1-Linoleoyl glycerol	67.05479,81.07028,95.08576		0.08	23.505
42	Oleamide	55.05497,57.07060,69.07047,83.08598		0.04	23.792
43	Monoolein	69.07042,81.07029,265.25189		0.25	23.963
44	1-Stearoylglycerol	57.07063,71.08613,95.08589		3.96	24.43
45	Erucamide	69.07045,83.08598,97.10146		2.35	25.13

**Table 2:** Scoring criteria for mouse dorsal condition.

Score	Characterization (Wrinkles)	Characterization (Injury)
1	Smooth skin, no wrinkles	Smooth skin
2	Fine lines	Slight nodule formation
3	A few shallow wrinkles	Moderate nodule formation
4	Numerous prominent wrinkles	Severe nodules with extensive hyperpigmentation
5	Thickened skin with deep wrinkles	Inflammation
